# Marine Natural Products from Tunicates and Their Associated Microbes

**DOI:** 10.3390/md19060308

**Published:** 2021-05-26

**Authors:** Chatragadda Ramesh, Bhushan Rao Tulasi, Mohanraju Raju, Narsinh Thakur, Laurent Dufossé

**Affiliations:** 1 Biological Oceanography Division (BOD), CSIR-National Institute of Oceanography (CSIR-NIO), Dona Paula 403004, India; 2 Department of Ocean Studies and Marine Biology, Pondicherry Central University, Brookshabad Campus, Port Blair 744102, India; mohanrajupu62@gmail.com; 3 Zoology Division, Sri Gurajada Appa Rao Government Degree College, Yellamanchili 531055, India; bhushanphd@gmail.com; 4 Chemical Oceanography Division (COD), CSIR-National Institute of Oceanography (CSIR-NIO), Dona Paula 403004, India; thakurn@nio.org; 5 Laboratoire de Chimie et Biotechnologie des Produits Naturels (CHEMBIOPRO), Université de La Réunion, ESIROI Agroalimentaire, 15 Avenue René Cassin, CS 92003, CEDEX 9, F-97744 Saint-Denis, Ile de La Réunion, France

**Keywords:** tunicates, symbiotic microbes, pigments, bioactive compounds, alkaloids &amp, peptides

## Abstract

Marine tunicates are identified as a potential source of marine natural products (MNPs), demonstrating a wide range of biological properties, like antimicrobial and anticancer activities. The symbiotic relationship between tunicates and specific microbial groups has revealed the acquisition of microbial compounds by tunicates for defensive purpose. For instance, yellow pigmented compounds, “tambjamines”, produced by the tunicate, *Sigillina signifera* (Sluiter, 1909), primarily originated from their bacterial symbionts, which are involved in their chemical defense function, indicating the ecological role of symbiotic microbial association with tunicates. This review has garnered comprehensive literature on MNPs produced by tunicates and their symbiotic microbionts. Various sections covered in this review include tunicates’ ecological functions, biological activities, such as antimicrobial, antitumor, and anticancer activities, metabolic origins, utilization of invasive tunicates, and research gaps. Apart from the literature content, 20 different chemical databases were explored to identify tunicates-derived MNPs. In addition, the management and exploitation of tunicate resources in the global oceans are detailed for their ecological and biotechnological implications.

## 1. Introduction

Tunicates and sea squirts are soft-bodied solitary or colonial (60%) sessile marine organisms belonging to the family Ascidiacea under the subphylum Urochordata, phylum Chordata [1,2]. These organisms are hermaphroditic, filter feeders, and appear in different body colors, such as translucent to blue, green, yellow, red, and brown, with a life span ranging from two months to one year [1,2,3,4]. Currently, tunicates are classified into four major clades such as (a) Appendicularia, (b) Thaliacea + Phlebobranchia + Aplousobranchia, (c) Molgulidae, and (d) Styelidae + Pyuridae, on the basis of the phylogenomic transcriptomic approach [5]. Globally, around 2815 tunicate species have been recorded from shallow coastal waters to deep waters [1]. Tunicate larvae resemble tadpole larvae of members of Chordata, but soon after the retrogressive metamorphosis, they lose the notochord and post-anal tail; thus, these organisms are often referred to as the “evolutionary connecting link” between invertebrates and chordates [6,7]. Therefore, tunicates are considered as important model organisms for several research aspects, such as evolution [6], development biology [8,9], invasion success [10], and bioactive compounds.

Tunicates are prolific producers of marine natural products (MNPs), and certain species are also known to release toxins, such as Bistramide A [11,12]. However, a few species, like *Halocynthia roretzi* and *Pyura michaelseni*, are eaten in southeast Asian countries like Korea [13,14]. The strong immune defensive system [15] and their associated symbiotic microbes with bioactive properties [16], makes tunicates highly preferential drug resources in the ocean [15,17]. Since the majority of the tunicate species are known to produce MNP’s, attempts are being undertaken in the culturing of these tunicates (e.g., mangrove tunicate *Ecteinascidia turbinata*) in large scale for various applications [18,19]. The process of accumulation of vanadium by vanadocytes of tunicates from seawater is well-known [20]. In contrast, investigations on the acquisition of MNPs by tunicates from their symbiotic bacteria are very limited, except for the antitumor products ecteinascidins [21,22], didemnin [23], and talaropeptides [24]. A recent review highlighted the association of bacteria, actinomycetes, fungi, and cyanobacteria with the tunicates and their bioactive nature [25]. It was also observed that actinomycetes, fungi, and bacteria are the predominant microbes associated with the tunicates, showing cytotoxic and antimicrobial activities [26], with the production of alkaloids as the major source of MNPs [27]. In this context, this review aimed to provide the chemical profiles of various tunicates and their associated microbes for biotechnological and drug development applications.

## 2. Ecological Importance of Tunicates

The tunicates population plays an important role in the marine food web through filter feeding [4]. Earlier studies have suggested that phytoplankton productivity in a shallow fjord is controlled by the tunicates population [28]. Tunicates are known to trap the sinking particulate organic matter and generate mucus rich organic matter and fecal pellets with carbohydrates and minerals [29,30], thereby triggering the downward biogeochemical flux (e.g., carbon flux) patterns from surface to deep waters [29,31,32]. Some obligate photosymbiotic tunicates have been suggested to act as environmental stress indicators [33]. The unknown ecological functions of a few tunicate MNPs [34] in understanding their ecological role is yet to be understood.

## 3. Database Search on Tunicate MNPs

Twenty different public chemical databases such as BIAdb, BindingDB, ChemDB, ChEMBL, ChemSpider, DrugBank, HIT, HMDB, KEGG, NCI, NPACT, PDB-Bind, PDBeChem, PharmaGKB, PubChem, SMPDB, SuperDrug, TTD, UNIProt, and ZINC were explored to identify the tunicate-originated MNPs deposited in these databases. The chemical constituents identified from these databases using the search keywords “tunicate and ascidian” are listed in Table 1.

## 4. Profile of MNPs from Tunicates and Associated Microbes

Tunicates are known to produce a wide range of MNPs with various bioactive properties (Table 2 and Table 3). These organisms are considered as a rich source of cellulose, which varies with different species [35]. Alkaloids and peptides are the major chemical constituents observed in tunicates [36]. Metabolites originated from tunicate hemocytes are also found to be cytotoxic to foreign particles [37] and various cell lines [38]. Microorganisms associated with the invertebrate hosts have also been identified as a source of bioactive metabolites [39]. In fact, bioactive metabolite-producing invertebrate-associated microorganisms have special implications in solving the “supply problem” in the initial steps of drug discovery [40]. Recently, Chen et al. reviewed the biological and chemical diversity of ascidian-associated microorganisms [41].

**Figure 1:**
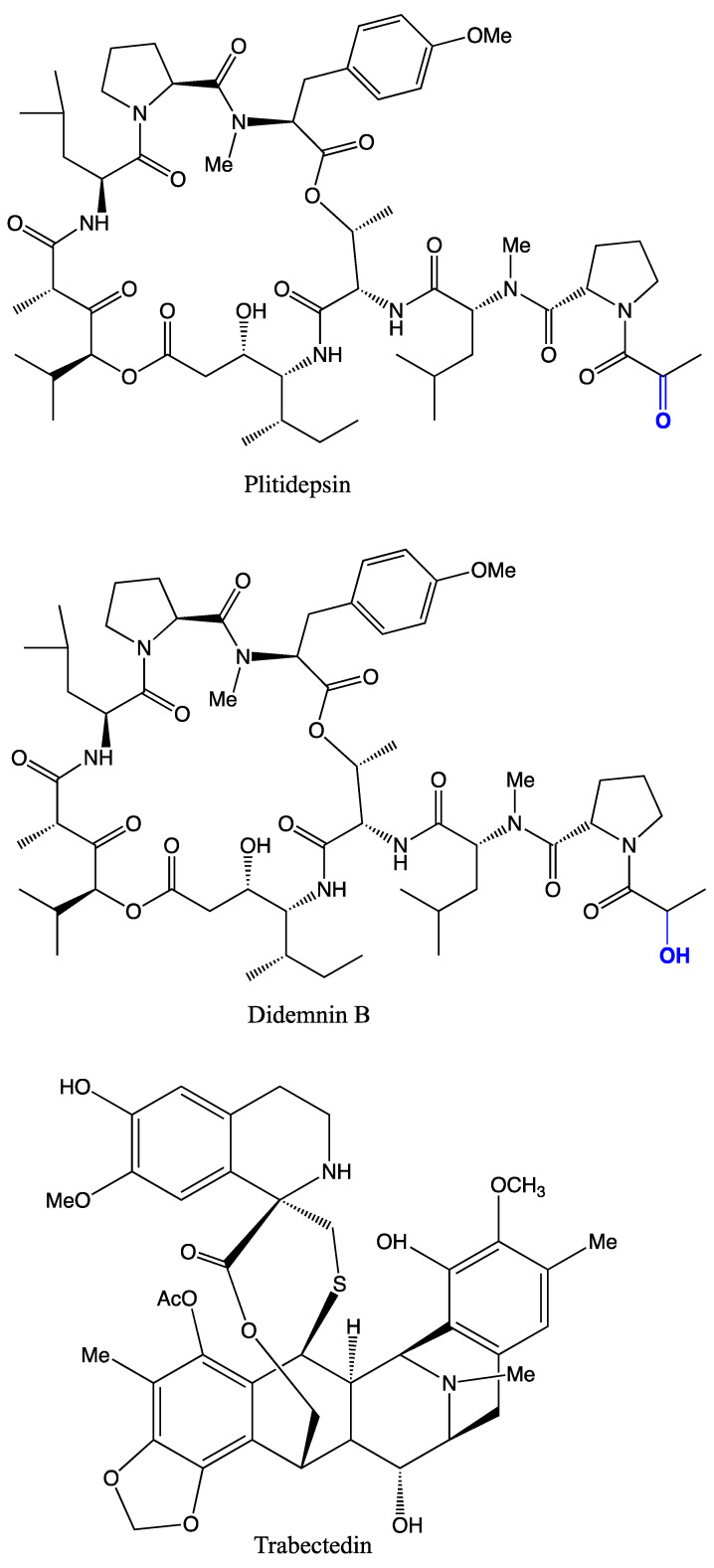
Important anticancer drugs of tunicates and their associated microbes in clinical trials.

**Figure 2:**
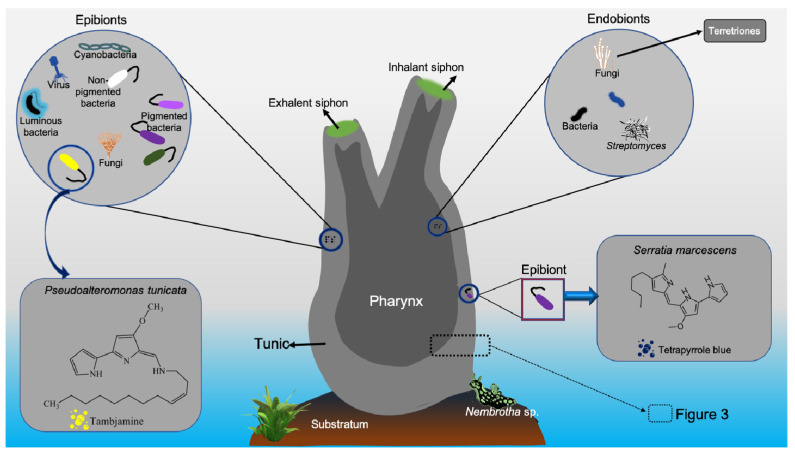
Tunicate-associated epibiotic and endobiotic symbionts. (the small inserted empty box provides more details in Figure 3).

**Figure 3:**
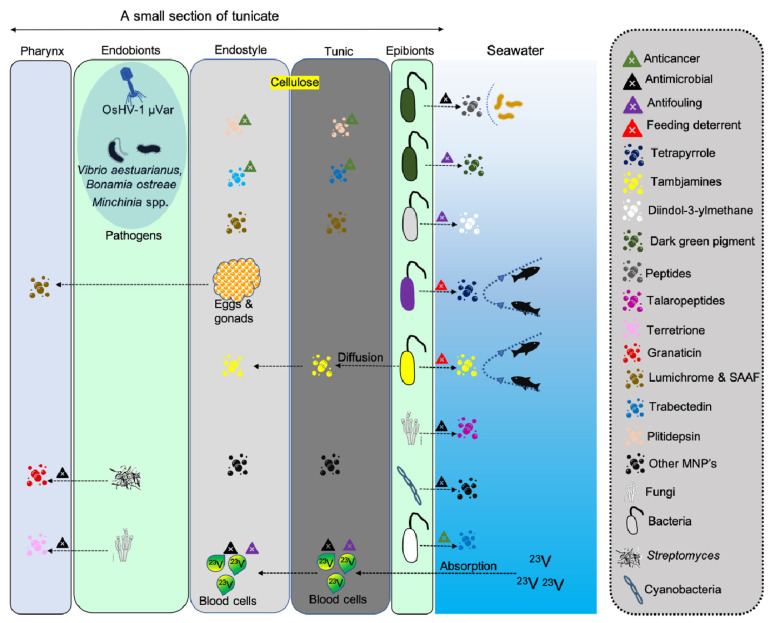
Illustration depicting various MNPs released from endobiotic and epibiotic microbes associated with tunicate’s endostyle and tunic.

Microbes associated with tunicates have been found to produce potential metabolites showing antimicrobial and anticancer activities (Figure 1, Figure 2 and Figure 3 and Table 3). Tunicate-associated bacteria such as *Bacillus*, *Pantoea*, *Pseudoalteromonas*, *Salinicola*, *Streptomyces*, *Vibrio* and *Virgibacillus* have recently been identified with potential antimicrobial activities [16]. The introduced tunicate species are also reported to harbor diverse host-specific microbial populations [49] that produce species-specific metabolites [50]. In general, tunicate associated bacteria and fungi are known to produce a variety of MNPs with various biological properties [41]. The chemistry of yellow pigment-producing parasitic bacteria in the interstitial and blood-filled spaces of planktonic tunicates, *Oikopleura vanhoeffeni* and *Oikopleura dioica*, are yet to be characterized [51].

## 5. Antimicrobial Applications

Tunicates [123], with their associated epi-symbionts [16,124] and endosymbionts [125], are prolific producers of antimicrobial and antifungal compounds inhibiting pathogens. The brominated alkaloids [126] and other compounds from tunicates have been reported to possess several biological activities [25,26]. *Pseudoalteromonas tunicata* produces alkaloid tambjamine (425 nm), an antifungal yellow pigment [127,128], and violacein (575 nm), a purple pigment with antiprotozoal activity [129,130], in addition to a range of bioactive compounds [129,131]. Methanol extraction of *Lissoclinum fragile* displayed antibacterial, antifungal, hemolytic, and cytotoxic activities [92]. The kuanoniamine A metabolite produced by *Eusynstyela tincta* inhibited pathogenic bacteria such as *B. subtilis, E. coli, S. aureus, V. cholerae*, and *V. parahaemolyticus* and fungi *A. fumigatus* and *C. albicans* [88]. A diffusible 190-kDa protein produced by tunicate *Ciona intestinalis* associated bacterium *Pseudoalteromonas tunicata* was found to show antibacterial activity against marine isolates [132]. The four α-helical peptides “clavanins A, B, C, and D” isolated from the hemocytes of tunicate *Styela clava* showed antibacterial activity against pathogenic *Listeria monocytogenes* strain EGD and antifungal activity against *Candida albicans* [44]. Halocidin, an antimicrobial peptide purified from tunicate *Halocynthia aurantium* showed antibacterial activity against methicillin-resistant *Staphylococcus aureus* and multidrug-resistant *Pseudomonas aeruginosa* [47]. Similarly, halocyntin and papillosin peptides isolated from tunicate *Halocynthia papillosa* also displayed antibacterial activity against both Gram-positive and Gram-negative marine bacteria [46]. Halocyamine peptides synthesized by the hemocytes of *Halocynthia roretzi* showed antimicrobial activity against various bacteria and yeasts [90]. Similarly, Halocyamines produced by *Styela clava* also displayed antimicrobial properties [108]. A salt-tolerant peptide isolated from hemocytes of *Ciona intestinalis* showed both antibacterial and antifungal activity [133]. Vanadium chloride and vanadyl sulfate also displayed antibacterial activity against various pathogens [95].

An endobiont, *Streptomyces* sp., isolated from the tunicate, *Styela canopus*, produced antibacterial compounds such as granaticin, granatomycin D, and dihydrogranaticin B [121]. Similarly endosymbiotic fungi associated with the tunicates, *Polycarpa aurata* [134] and *Rhopalaea crassa* [135], showed antimicrobial activity. The fungi *Talaromyces* sp., isolated from an unidentified tunicate, produced talaropeptides A and B, two antibacterial metabolites that inhibited Gram-positive bacteria, *Bacillus subtilis* [24]. The endophytic fungus *Penicillium* sp. isolated from *Didemnum* sp. produced antifungal and cytotoxic compounds, terretrione C and D [136].

Some tunicates produced antiviral molecules, indicating their chemical defense function against environmental viruses. The Caribbean tunicate, *Trididemnum* sp., was found to produce depsipeptides, particularly didemnin A and B, exhibiting antiviral activity against DNA and RNA viruses in vitro [111,137]. Another species of Caribbean tunicate, *Eudistoma olivaceum*, produced prolific MNPs, such as eudistomins A, D, G, H, I, J, M, N, O, P, and Q, which possessed antiviral activity [83]. The ascidian *Didemnum guttatum* was found to produce the cyclodidemniserinol trisulfate compound that showed anti-retroviral activity by inhibiting HIV-1 integrase [72]. The tunicate, *Didemnum molle*, released lanthipeptide divamide A that promised to be a potential anti-HIV drug [74] (Table 4).

## 6. Anticancer and Antitumor Applications

Trabectedin (Ecteinascidin; ET-743; Yondelis^®^), an alkaloid extracted from the orange tunicate, *Ecteinascidia turbinata*, is approved as a first anticancer drug [138] to treat breast cancer [139,140], soft tissue sarcoma [141], and ovarian cancer [142,143,144]. This molecule is suggested to originate from *E. turbinata* symbiotic bacteria, *Candidatus Endoecteinascidia frumentensis* [145]. However, plitidepsin (Aplidin^®^), a depsipeptide isolated from the Mediterranean tunicate, *Aplidium albicans*, is in phase II clinical trials [138,146] as an anticancer drug against breast cancer [147], human kidney carcinoma cells [52], and multiple myeloma [53]. Didemnin B is also in phase II trials [148], showing anticancer activity against leukaemia P388 cells [111]. Significantly, 60% of the human cervical carcinoma cell lines (HeLa) were inhibited by Eudistomins H extracts (IC_50_ 0.49 μg/mL) obtained from *E. viride* [86]. Clavepictine A and B alkaloids originated from *Clavelina picta* demonstrated potential cytotoxic activity (IC_50_ 12 μg/mL) against murine leukemia and human solid tumor cell lines [62]. Lamellarin sulfates originated from *Didemnum ternerratum* [78] and polycarpine dihydrochloride, a disulfide alkaloid extracted from an ascidian *Polycarpa clavata*, were found to inhibit human colon tumor cell lines [97].

Cystodytins A, B, and C, three teracyclic alkaloids isolated from Okinawa tunicate *Cystodytes dellechiajei*, were reported to show antitumor activities [64]. Macrolides isolated from tunicates *Lissoclinum patella* (Patellazole C) [94] and *Eudistoma* cf. *rigida* (Lejimalides A, B, C, and D) [149,150] possessed anticancer activity [151]. Diplamine, an orange pigment alkaloid produced by *Diplosoma* sp., demonstrated cytotoxic activity against leukemia cells [79]. Halocyamine A and B peptides extracted from *H. roretzi* showed anticancer activity against various cell lines [90]. A depsipeptide, dehydrodidemnin B, produced by *Aplidium albicans* inhibited Ehrlich carcinoma cells in mice and reduced 80–90% tumor cells [54]. Bryostatins Ecteinascidins products, such as ET-729, 743, 745, 759 A, 759B, and 770, extracted from the Caribbean tunicate *Ecteinascidia turbinata* showed immunomodulator activity and antitumor activity against various leukemia cells [152] and breast, lung, ovary, and melanoma cells [153]. The Brazilian ascidian, *Didemnum granulatum*, produced G2 checkpoint-inhibiting aromatic alkaloids, granulatimide and isogranulatimide [154]. The ascidian *Cystodytes dellechiajei* produced topoisomerase II-inhibiting ascididemin, which has antitumor activity against various tumor cell lines [66]. This marine alkaloid exhibits marked cytotoxic activities against a range of tumor cells. The kuanoniamine A metabolite extracted from *E. tincta* displayed 100% inhibition of Dalton’s lymphoma and Ehrlich ascites tumor cell lines [88]. Cynthichlorine, an alkaloid isolated from the tunicate *Cynthia savignyi*, showed cytotoxicity against *Artemia salina* larva at an LD_50_ of 48.5 μg/mL [63]. Siladenoserinols A and B derivatives isolated from didemnid tunicates possessed antitumor activity by inhibiting the interaction of p53-Hdm2 [69] (Table 4).

## 7. Antifouling and Anti-Deterrent Activities

The colonial tunicate, *Eudistoma olivaceum*, was found to produce brominated alkaloids, Eudistomins G and H, which acted as antifouling substances and fish antifeedants; thus, the *E. olivaceum* surface was completely free from fouling epibionts [34]. A dark green pigmented bacteria, *Pseudoalteromonas tunicata*, isolated from the surface of *Ciona intestinalis*, collected originally from off the west coast of Sweden, showed antifouling activity against algal spores, invertebrate larvae, and diatoms [131,155,156]. The yellow pigmented *Pseudoalteromonas tunicata* mutants have demonstrated antifouling activity against algal spore germination, bacterial growth, fungal growth, and invertebrate larvae [129]. Diindol-3-ylmethane products isolated from an unidentified ascidian-associated bacteria, *Pseudovibrio denitrificans*, displayed nearly 50% antifouling activity against barnacle *Balanus amphitrite* and bryozoan *Bugula neritina* [118].

Deterring activity of vanadium acidic solutions, such as vanadyl sulfate and sodium vanadate, was observed against *Thalassoma bifasciatum* when incorporated into food pellets [95,157]. Didemnimides C and D from *Didemnum conchyliatum* [158], nordidemnin B [102] and didemnin B [159] from *Trididemnum solidum*, and granulatamides from *Didemnum granulatum* [73] displayed antifeedant effects on various fishes in laboratory experiments. The kuanoniamine A molecule from *E. tincta* displayed feeding-deterrent activities against carnivore gold fish, *Carassius auratus* [88]. MNPs isolated from Antarctic tunicates have demonstrated variability in anti-deterrent activities [58]. Both the yellow pigmented tambjamine metabolites and blue tetrapyrrole metabolite released from *Sigillina* sp. (i.e., *Atapozoa* sp.) showed feeding-deterrent activity against various carnivore fishes [59,160]. The blue tetrapyrrole pigment was suggested to originate from the associated bacteria *Serratia marcescens* [120]. Tambjamines and tetrapyrrole chemical constituents from both adult and larvae were reported to function as defensive chemicals against predators [102]. Lipophilic crude extracts from Antarctic tunicate, *Distaplia cylindrica* [161], and polyandrocarpidines from *Polyandrocarpa* sp. [101,102] demonstrated deterrent activity against certain sea-stars, hermit crabs, and snails (Table 4).

## 8. Miscellaneous Applications

The chiton *Mopalia* sp. spawned when injected with 1.0 mg/L of gonadotropin releasing hormone (GnRH2) of a tunicate [48]. Lumichrome, a compound extracted from tunic, gonads, and eggs of ascidian, *Halocynthia roretzi*, was involved in the larval metamorphosis [89]. Similarly, sperm-activating and attracting factors (SAAF) were isolated from eggs of the ascidians *Ciona intestinalis* and *Ascidia sydneiensis* [162]. Lipids extracted from *H. roretzi* have demonstrated the antidiabetic and anti-obese properties in mice models [163]. Two novel alkaloids, mellpaladine and dopargimine, isolated from Palauan tunicate have demonstrated neuroactive behavior in mice [68]. Two new alkaloids, polyaurines A and B, isolated from the tunicate, *Polycarpa aurata*, inhibited blood-dwelling *Schistosoma mansoni* [96]. Lepadin and villatamine alakaloids isolated from *Clavelina lepadiformis* [61] and lepadins from *Didemnum* sp. [71] displayed potential antiparasitic and cytotoxic activities. The ascidian species, *Didemnum psammathodes*, collected from the central west coast of India was extracted in organic solvents. These extracts showed antimicrobial and antifouling properties [164].

## 9. Issues in Extraction & Identification of Tunicate MNPs

Marine organisms have developed diverse secondary metabolic pathways, which produce a vast number of unusual chemical moieties. These compounds belong to a wide variety of chemical classes, including terpenes, shikimates, polyketides, peptides, alkaloids, and many unidentified and uncharacterized structures (Houssen and Jaspars, 2012). There are several technologies in place to isolate and characterize the natural products from even a very small quantity of marine organisms. However, there are still hurdles in the isolation and characterization of bioactive molecules from ascidians. These include 1. taxonomic uncertainty: worldwide, there are very few taxonomists available for proper taxonomic assignments of tunicates. Sometimes the identification using molecular tools has been complicated by the difficulty in getting pure gDNA from the target species due to complex biotic associations (Houssen amd Jaspars, 2012). 2. Quantity of isolated molecules: most of the time, a small quantity of metabolites is available in the organisms, which is not even sufficient for spectroscopic analysis. 3. Instability of molecules: there are extremely labile compounds in the extracts, which decompose during the purification process, and we get artefacts. Of course, these problems are common in other marine invertebrates as well. Research funding has also become a hurdle for many young researchers; thus, many researchers are publishing their works with crude extracts instead of analyzing complete structural elucidation. If we could address these issues, we will be able to isolate and characterize novel bioactive molecules from this unique group of marine invertebrates. The quantity of molecules can be increased if we collect the target tunicate species at the right time (season) from the correct geographic location. This can be achieved by understanding the chemical ecology of the producing species. For this purpose, there should be joint efforts from marine biologists, ecologists, and natural product chemists.

## 10. Metabolic Origin of Some Tunicates and Their Predators

Several bioactive MNPs extracted from tunicates were believed to be originated from tunicates themselves. However, few studies have investigated the original origin of tunicate MNPs from their symbiotic microbes. Tambjamine pigments have been reported to be originated from tunicate-associated symbiotic bacteria like *S. marcescens* [160] and *Pseudoalteromonas tunicata* [116,131]. An identical dark blue pigmented tetrapyrrole compound isolated from an ascidian was observed from a bacterium [165]. The blue tetrapyrrole pigment was reported to have originated from the associated bacteria, *Serratia marcescens* [120]. Didemnins extracted from the tunicate, *T. solidum* [111], are found to be released by associated bacteria, *Tistrella mobilis* and *Tistrella bauzanensis* [23,122]. Similarly, the trabectedin compound identified from the Caribbean tunicate, *E. turbinata* [152,166], has now been observed to be produced by its symbiotic bacteria, *Candidatus Endoecteinascidia frumentensis* [145]. Meridianins isolated from Antarctic tunicates, *Aplidium, Synoicum*, and some sponges, are thought to have originated from their symbiotic microbes [58]. Similarly, tetrahydroisoquinoline constituents identified from the tunicate, *Ecteinascidia turbinata*, appeared to be released by the unculturable endosymbiotic bacterium, *Candidatus Endoecteinascidia frumentensis* [113]. Some of the bioactive MNPs identified from Didemnid tunicates also originated from their symbiotic cyanobacterial species, such as *Synechocystis* and *Prochloron* [167,168]. Namenamicin produced by the orange color ascidian, *Polysyncraton lithostrotum*, was suggested to originate from its symbiotic bacterium, *Micromonospora* species [100]. The anti-HIV lanthipeptide, divamide A, isolated from the tunicate, *Didemnum molle*, was found to be produced by uncultivable symbiotic bacteria [74].

Tunicates are known to produce more than 300 alkaloid compounds [126]. The tunicate predatory flatworm *Prostheceraeus villatus* was reported to obtain alkaloids, lepadins, and villatamines by preying (dietary origin) on the tunicate, *Clavelina lepadiformis* [61]. Likewise, tambjamine alkaloids observed in the ascidian *Atapozoa* sp. [160] and associated bacteria [131] were found to be acquired by the predatory nudibranchs, like *Nembrotha* sp., for defense functions [59,169]. Pyridoacridine metabolites observed in ascidians and some sponges indicate a possible microbial origin or convergent evolution of these molecules [170].

## 11. Utilization of Invasive Tunicates Resources

Tunicates usually occur in relatively low abundance in coastal waters. However, some tunicates are reported as invasive species in some coastal waters [171] and are known to cause space competition [172], damage to aquaculture [173,174] by harboring pathogenic viruses and bacteria [175], and ecosystem alteration within the spread area [176]. Few non-invasive tunicate species of the coral reef environment have also been reported to overgrow on massive corals and caused minimal [112] or partial inhibition or delayed development of coral polyps [177]. A study reported the outbreak of the invasive tunicate, *Diplosoma similis*, that overgrew on corals and macrophytes and resulted in 50% mortality of corals [178] (Table 5).

Therefore, such overwhelming invasive species may be utilized to investigate their biological properties, biotechnological implications, and drug development. The exploitation of antiviral and cytotoxic didemnins from the invasive tunicate, *T. solidum*, has already been investigated [111,112]. Antimicrobial activity of α-helical peptides “Clavanins” was identified from the hemocytes of the tunicate, *Styela clava* [44]. Thus, other invasive species need to be investigated for their bioactive properties. Seasonal studies on the spread of various invasive tunicates and their biomass estimations are an important research aspect for resource management and coastal conservation. A study suggested that ocean warming is triggering the rise of invasive species in coastal waters [185]. Therefore, identifying the key ocean-warming factors and their mitigation strategies is essential for a sustainable management of the global ocean bioresources.

## 12. Research Gaps and Future Perspective

Tunicates have been an important marine drug reservoir to treat a variety of diseases, including cancer. These resources from the ocean, particularly from the deep-sea, remain untapped for drug discovery. Therefore, exploration and exploitation of tunicate resources from coastal waters to the deep-sea and tropical to polar regions would open new insights in the drug discovery and evolutionary lineages. However, these efforts should be driven by chemical ecology of these organisms. The study of chemical ecology will help in bioprospecting and the efficient production of marine drugs from this unique group of organisms. On the other hand, the mode of colonization and pigment biosynthesis by associated microbes and the acquisition mechanism of pigments (e.g., tambjamines) by tunicates from their associated microbes are yet to be unveiled. Since tunicates have been reported to be colonized by pathogenic bacteria during filter feeding, the pathological implications of tunicates needs to be investigated to understand the possible transfer ways of pathogenic bacteria from tunicates to other biota and aquaculture setups. Therefore, regular biodiversity monitoring and population dynamics of tunicate resources should be performed to understand their distribution patterns and impact on the coastal resources.

## Figures and Tables

**Table 1:** List of MNPs originated from tunicates available in various public databases. The unknown compound records are excluded from the list.

Database	No. of Known Compounds	No. of Unknown Compounds	Known Chemical Compound	Biological Properties
BIAdb	1	-	Polycarpine	Cytotoxic, antiviral, and antifungal
BindingDB	2	-	Tuberatolides, Sodium 1-(12-hydroxy) octadecanyl sulfate	Farnesoid X receptor antagonists, matrix metalloproteinase 2 inhibitor
ChemDB	2	-	Patellazole B, Patellazole C	Antimicrobial, cytotoxic
ChEMBL	2		Ascididemin, Trabectedin	Anticancer
ChemSpider	1	-	Trabectedin	Anticancer
DrugBank	-	-		
HIT	-	-		
HMDB	1	-	Trabectedin	Anticancer
KEGG	1	-	Trabectedin	Anticancer
NCI	-	-		
NPACT	-	-		
PDB-Bind	-	-		
PDBeChem	16	>30	Cystodytin D, cystodytin F, cystodytin E, cystodytin G, cystodytin H, cystodytin I, Diplosoma ylidene 1, Diplosoma ylidene 2, Lejimalide A, lejimalide B, lissoclibadin 1, lissoclibadin 2, lissoclibadin 3, lamellarin alpha 20-sulfate, plitidepsin, trabectedin	Cytotoxic, anticancer
PharmaGKB	1	-	Trabectedin	Anticancer
PubChem	4	2	Patellazole B, Patellazole C, GnRH-II, GnRH-I	Antimicrobial and cytotoxic, induces spawning
SMPDB	-	-		
SuperDrug	1	-	Trabectedin	Anticancer
TTD	-	-		
UniProt	1	1	Retinoic acid	Regeneration of gut
ZINC	1	-	Trabectedin	Anticancer

**Foot note:** Table 1 data are garnered from public chemical databases listed in the main text part 3, but not from the literature. That is why there are no references cited in this table. Readers are asked to refer to Tables 2 and 3 where details are from the literature, and therefore, references are cited.

**Table 2:** Chemical profiles from body parts and fluids of few tunicate species.

Body Component	Chemical Compound	Function	Application	Reference
Tunic (*Ascidia* sp., *Ciona intestinalis, Halocynthia roretzi*, and *Styela plicata*)	Tunicin (cellulose)	Protection	Material cellulose	[35]
Blood (*Ascidia nigra*, *Molgula manhattensis*)	Vanadium	Oxygen transport		[42]
Blood (*Ascidia nigra*)	Tunichromes	Vanadium binding and reduces blood pigments		[42,43]
Hemocytes (*Styela clava*)	Clavanins	Multiple functions	Antimicrobial	[44,45]
Hemocytes (*Halocynthia papillosa*)	Halocyntin and papillosin		Antimicrobial	[46]
Hemocytes (*Halocynthia aurantium*)	Halocidin		Antimicrobial	[47]
Gonad (Unknown sp.)	GnRH-2 peptide	Pheromone-like function	Induce spawning	[48]

**Table 3:** Bioactive compounds from various species of tunicates and their associated microbes.

MNPs from Tunicates	Chemical Compound	Function	Application	Reference
*Aplidium albicans*	Aplidin		Anticancer	[52,53]
*Aplidium albicans*	Dehydrodidemnin B		Antitumor	[54]
*Aplidium glabrum*	Quinones		Anticancer, cytotoxic	[55]
*Aplidium haouarianum*	Haouamine A		Cytotoxic activity	[56]
*Aplidium meridianum*	Meridianins		Anticancer, antibiofilm	[57]
*Aplidium* & *Synoicum*	Meridianins	Feeding deterrents	Antibacterial	[58]
*Atapozoa* sp.	Tambjamine	Feeding deterrents		[59]
*Botryllus tuberatus*	Tuberatolides		Farnesoid X receptor antagonists	[60]
*Clavelina lepadiformis*	Lepadins and villatamines		Antiparasitic, anticancer	[61]
*Clavelina picta*	Clavepictine A and B		Antimicrobial, cytotoxicity	[62]
*Cynthia savignyi*	Cynthichlorine		Antifungal, cytotoxicity	[63]
*Cystodytes dellechiajei*	Cystodytins A-I		Antitumor, cytotoxic	[64,65]
*Cystodytes dellechiajei*	Ascididemin		Antitumor	[66]
*Cystodytes* sp.	Ascididemin	Feeding deterrents	Antifeedant	[67]
Didemnidae	Mellpaladine and dopargimine		Neuroactive	[68]
Didemnidae	Siladenoserinols A and B		Antitumor	[69]
Didemnidae	Sameuramide A		Colony formation	[70]
*Didemnum* sp.	Lepadins D-F		Antiplasmodial and antitrypanosomal	[71]
*Didemnum guttatum*	Cyclodidemniserinol trisulfate		Anti-retroviral	[72]
*Didemnum granulatum*	Granulatamides		Deterrent activity	[73]
*Didemnum molle*	Lanthipeptide divamide A		anti-HIV drug	[74]
*Didemnum molle*	Mollamide B		Anticancer	[75]
*Didemnum proliferum*	Shishijimicins		Antitumor	[76]
*Didemnum psammatodes*	Methyl esters		Antiproliferative	[77]
*Didemnum ternerratum*	Lamellarin Sulfates		Anticancer	[78]
*Diplosoma* sp.	Diplamine		Antibacterial and cytotoxic	[79]
*Diplosoma virens*	Diplosoma ylidene 1, Diplosoma ylidene 2		Anticancer	[80]
*Ecteinascidia turbinata*	Ecteinascidin 743 (Trabectedin)		Anticancer	[81]
*Eudistoma gilboverde*	Methyleudistomins		Antitumor	[82]
*Eudistoma olivaceum*	Eudistomins G and H	Chemical defense	Antifouling	[34]
*Eudistoma olivaceum*	Eudistomins A, D, G, H, I, J, M, N, O, P, and Q		Antiviral	[83]
*Eudistoma olivaceum*	Eudistomins C, E, K, and L		Antiviral	[84]
*Eudistoma vannamei*	7-Oxostaurosporine		Anticancer	[85]
*Eudistoma viride*	Eudistomins H		Anticancer	[86]
*Eusynstyela latericius*	Eusynstyelamides A, B		Antibacterial	[87]
*Eusynstyela tincta*	Kuanoniamine A	Chemical defense	Antimicrobial, antitumor, antifouling	[88]
*Halocynthia aurantium*	Halocidin		Antimicrobial	[47]
*Halocynthia papillosa*	Halocyntin and papillosin		Antimicrobial	[46]
*Halocynthia roretzi*	Lumichrome	Larval metamorphosis		[89]
*Halocynthia roretzi*	Halocyamine A and B		Antimicrobial, anticancer	[90]
*Lissoclinum* cf. *badium*	Lissoclibadins		Anticancer	[91]
*Lissoclinum fragile*			Antimicrobial, hemolytic, and cytotoxic	[92]
*Lissoclinum patella*	Patellazole B and C		Antimicrobial, cytotoxic	[93,94]
*Phallusia nigra*	Vanadium chloride, vanadyl sulfate		Antimicrobial	[95]
*Polycarpa aurata*	Polyaurines A and B		Antiparasitic	[96]
*Polycarpa clavata*	Polycarpine dihydrochloride		Cytotoxic	[97]
*Polycarpa clavata*	Polycarpaurines A and C		Antiviral, antifungal	[98]
Polyclinidae	Sodium 1-(12-hydroxy) octadecanyl sulfate		Matrix metalloproteinase 2 inhibitor	[99]
*Polysyncraton lithostrotum*	Namenamicin		Cytotoxic, antitumor	[100]
*Polyandrocarpa* sp.	Polyandrocarpidines		Antimicrobial, cytotoxic, and deterrent activities	[101,102]
*Polyandrocarpa misakiensis*	Retinoic acid		Regeneration of gut	[103]
*Pseudodistoma antinboja*	Cadiolides J-M		Antibacterial	[104]
*Pycnoclavella kottae*	Kottamide D		Cytotoxic, anti-inflammatory, and antimetabolic activities	[105]
*Sidnyum turbinatum*	Alkyl sulfates		Antiproliferative	[106]
*Stolonica* sp.	Stolonic acid A and B		Antiproliferative	[107]
*Styela clava*	Clavanins		Antimicrobial	[108]
*Styela plicata*	Hemocytes		Cytotoxic	[109]
*Synoicum adareanum*	Hyousterones and Abeohyousterone		Cytotoxic and anticancer	[110]
*Trididemnum solidum*	Didemnins A, B, and C		Antiviral, cytotoxic	[111,112]
**MNPs from associated microbes**				
*Candidatus* Endoecteinascidia frumentensis	Tetrahydroisoquinoline			[113]
*Microbulbifer* sp.	Bulbiferates A and B		Antibacterial	[114]
*Penicillium verruculosum*	Verruculides A, chrodrimanins A and H		Protein tyrosine phosphatase 1B inhibition	[115]
*Pseudoalteromonas rubra*	Isatin	Microbial defense	Antibacterial	[16]
*Pseudoalteromonas tunicata*	Tambjamine	Feeding deterrents		[116]
*Pseudoalteromonas tunicata*	Tambjamine		Antifungal	[117]
*Pseudovibrio denitrificans*	Diindol-3-ylmethanes		Antifouling	[118]
*Saccharopolyspora* sp.	JBIR-66		Cytotoxic	[119]
*Serratia marcescens*	Tetrapyrrole pigment	Feeding deterrents		[120]
*Streptomyces* sp.	Granaticin, granatomycin D, and dihydrogranaticin B		Antibacterial	[121]
*Talaromyces* sp.	Talaropeptides A-D		Plasma stability, Antibacterial, antifungal, cytotoxic	[24]
*Tistrella mobilis* and *Tistrella bauzanensis*	Didemnin		Anticancer	[23,122]

**Table 4:** Bioactive MNP’s from tunicates and associated microbes.

Application	Compound	Activity against	Dose/ Concentration	Growth Inhibition (Diameter/ Percentage)	Assay Method	Reference
**Antimicrobial**						
	Clavanins	*E. coli*, *L. monocytogenes*, *C. albicans*	1.6–3.5 μg/mL	-	Radial diffusion assay	[44]
	Diplamine	*E. coli*, *S. aureus*			-	[79]
	Halocidin	Methicillin-resistant *Staphylococcus aureus* and multidrug-resistant *Pseudomonas aeruginosa*	100–200 μg/mL	5–11 mm	Radial diffusion assay	[47]
	Isatin	*Bacillus cereus*, *Bacillus megaterium, Escherichia coli*, *Micrococcus luteus*,	MIC 200 μg/mL	7–>21 mm	Disk diffusion assay	[16]
	Kuanoniamine A	*B. Subtilis*, *E. coli*, *S. aureus*, *V. cholerae*, *V. parahaemolyticus* and fungus *A. jumigatus* and *C. albicans*	25 μg/mL	7–13 mm	Disk diffusion assay	[88]
	Cynthichlorine	*A. radiobacter*, *E. coli*, *P. aeruginosa*, *Botrytis cinerea*, *Verticillium albo atrum*		6–10 mm	Disc diffusion assay	[63]
	Talaropeptides A and B	*Bacillus subtilis*	IC_50_ 1.5–3.7 µM	50%	Microtiter plate assay	[24]
	Terretrione C and D	*Candida albicans*	MIC 32 µg/mL	17–19 mm	Disc diffusion assay	[136]
**Anticancer & antitumor**						
	Aplidin	Multiple myeloma cell lines, MDA-MB-231 breast cancer cells, A-498 and ACHN cell lines	IC_50_ 1 to 15 nmol/L		Nuclear Staining Assay; MTT assay	[52,53]
	Clavepictines A and B	Murine leukemia and human solid tumor cell lines	IC_50_ 12 μg/mL		Microculture tetrazolium assay	[62]
	Dehydrodidemnin B	Ehrlich carcinoma cells	2.5 μg/mouse	70–90%	MTT assay	[54]
	Didemnins A and B	Leukaemia P388 cells	IC_50_ 1.5–25 μg/mL		-	[111]
	Diplamine	Leukemia L1210 cells	IC_50_ 2×10^-2^ μg/mL		-	[79]
	Ecteinascidin 743 (Trabectedin)	Leukemia L1210 cells	IC_50_ 0.5 μg/mL		-	[152]
	Eudistomins H	HeLa cell lines	IC_50_ 0.49 μg/mL	60%	MTT assay	[86]
	Halocyamine A and B	Rat neuronal cells, mouse neuroblastoma N-18 cells, and human Hep-G2 cells			-	[90]
	Kuanoniamine A	Dalton’s lymphoma and Ehrlich ascites tumour cell lines	25 μg/mL	100%	Trypan blue exclusion test	[88]
	Lamellarin Sulfates	HCT-116 human colon tumor cells	IC_50_ 9.7 μM		MTS cell proliferation assay	[78]
	Namenamicin	P388 leukemia cells, 3Y1, and HeLa	IC_50_ 3.5 nM; IC_50_ 3.3–13 pM		Biochemical prophage induction assay	[100]
	Polycarpine dihydrochloride	HCT-116 human colon tumor cells	ED_50_ 1.9 μg/mL		-	[97]
	7-oxostaurosporine	HL-60, Molt-4, Jurkat, K562, HCT-8, MDA MB-435, and SF-295 cell lines	IC_50_ 10–58 nM	95%	MTT assay	[85]
	Terretrione C and D	Human breast cancer cells	IC_50_ 16.5 and 17.6 μM		Sulforhodamine B assay	[136]
**Antifouling**						
	Diindol-3-ylmethanes	Barnacle, *Balanus amphitrite* and bryozoan, *Bugula neritina*	EC_50_ 18.57		Microtiter plate assay	[118]
	Eudistomins G and H	Fish and other larvae			Antifeedant assay	[34]

**Table 5:** Occurrence of invasive tunicate species in the global ocean and their impact on the marine ecosystem.

Invasive Tunicate	Country	Origin Type	Negative Impacts	Reference
*Ascidiella aspersa*	Argentina	Exotic	Space competition	[179]
*Botrylloides violaceus*	Netherlands	Exotic	Space competition	[172]
*Botryllus schlosseri*	Netherlands	Indigenous	Space competition	[172]
*Botryllus schlosseri*, *Botrylloides violaceus*, *Ciona intestinalis*, *Ciona savignyi*, *Didemnum vexillum*, *Molgula manhattensis*, *Styela clava*	USA	Exotic	Competitors for food and space	[180,181]
*Ciona intestinalis*	Canada	Exotic	Mussel mortality	[176]
*Ciona intestinalis*	Korea	Exotic	Space competition and damage to aquaculture	[174]
*Didemnum psammathodes*	India	Indigenous	Space competition	[182]
*Didemnum vexillum*	USA	Exotic	Threat to eelgrass	[183]
*Didemnum vexillum*	Wales	Exotic	Space competition	[184]
*Diplosoma similis*	American Sāmoa	Indigenous	Kill corals	[178]
